# HOW THE IMPACT OF CHRONIC PAIN ON COGNITION VARIES BY POLYGENIC RISK SCORE (PRS)

**DOI:** 10.1093/geroni/igz038.3521

**Published:** 2019-11-08

**Authors:** Jieun Song

**Affiliations:** University of Wisconsin-Madison, Institute on Aging & Waisman Center, Madison, Wisconsin, United States

## Abstract

While prior research has found associations between chronic pain and cognition and genetic risk of cognitive decline, little research examined moderating effects of genetic risk on the association between chronic pain and cognition. This study investigate whether genetic risk of accelerated cognitive decline, assessed by polygenic risk score (PRS) of Alzheimer disease (AD), moderates the association between severe chronic pain and cognitive decline. The data are drawn from Midlife in the US (MIDUS), a survey of a nationally representative sample of US adults. The analytic sample consists of two groups: 201 individuals who reported severe chronic pain (116 women, 85 men) and 404 individuals without severe chronic pain (215 women, 189 men) who completed MIDUS 2 (2004-06) and MIDUS 3 (2013-14) surveys and participated in biomarker data collection. The findings showed that men who suffered from severe chronic pain were more vulnerable to genetic risk of cognitive decline than men who did not experience severe chronic pain. Specifically, men who suffered from severe chronic pain and had higher level of PRS of AD experienced a greater decline of episodic memory than men who experienced chronic pain with lower level of PRS of AD. This association was not found in women sufferers. For both men and women who did not have chronic pain, cognitive change was not a function of the level of genetic risk of cognitive decline. Findings suggest that genetic risk of cognitive decline would be manifested contingent on life circumstances as well as gender of individuals.

